# Correction: Diabetes and Overexpression of proNGF Cause Retinal Neurodegeneration via Activation of RhoA Pathway

**DOI:** 10.1371/journal.pone.0096960

**Published:** 2014-04-29

**Authors:** The PLOS ONE Staff

The authors would like to correct errors in the figures of the article.

In the Figure 2A for this article, we inadvertently included a duplicate of the control micrograph shown in Figure 5A in our previous publication (Mol Vis. 2012;18:2993-3003).

Figure 4C (proNGF) in the article corresponds to an image in Figure 5F in our publication in Diabetologia (Diabetologia (2013), 56: 2329 - 2339).

The authors apologize for these errors and are providing corrected figures for Figure 2 and Figure 4.

PLOS ONE requires that all content is published under the Creative Commons Attribution License (CC BY). The original images above were not published under this license and as a result, we have removed the previous Figure 2A and 4C from this article and replaced them by the corrected figures.
